# Project-based learning course on metabolic network modelling in computational systems biology

**DOI:** 10.1371/journal.pcbi.1009711

**Published:** 2022-01-27

**Authors:** Thomas Sauter, Tamara Bintener, Ali Kishk, Luana Presta, Tessy Prohaska, Daniel Guignard, Ni Zeng, Claudia Cipriani, Sundas Arshad, Thomas Pfau, Patricia Martins Conde, Maria Pires Pacheco

**Affiliations:** Systems Biology Group, Department of Life Sciences and Medicine, University of Luxembourg, Belvaux, Luxembourg; SIB Swiss Institute of Bioinformatics, SWITZERLAND

## Abstract

Project-based learning (PBL) is a dynamic student-centred teaching method that encourages students to solve real-life problems while fostering engagement and critical thinking. Here, we report on a PBL course on metabolic network modelling that has been running for several years within the Master in Integrated Systems Biology (MISB) at the University of Luxembourg. This 2-week full-time block course comprises an introduction into the core concepts and methods of constraint-based modelling (CBM), applied to toy models and large-scale networks alongside the preparation of individual student projects in week 1 and, in week 2, the presentation and execution of these projects. We describe in detail the schedule and content of the course, exemplary student projects, and reflect on outcomes and lessons learned. PBL requires the full engagement of students and teachers and gives a rewarding teaching experience. The presented course can serve as a role model and inspiration for other similar courses.

## Introduction

In contrast to teacher-centred teaching, project-based learning (PBL) is a dynamic student-centred teaching method that encourages the students to learn and solve relevant real-life problems on their own with the help of the teacher. Normally, in PBL, students start from a problem to be solved and organise themselves into a variety of activities, applying knowledge and techniques already acquired in a subject area, even though the aims can be broader. The project is usually time boxed and culminates with a report or oral presentation on the addressed problem. During the process, the teaching staff is engaged in trying to advise and motivate students, rather than in lecturing. A key advantage of PBL is that students are fully involved in the learning process while also improving their communication and collaboration skills [1,2].

In the area of computational biology, hands-on sessions and PBL activities are known to be one of the most effective ways to learn and disseminate skills [3]. PBL in computational biology provides the opportunity to face real-world scientific challenges while developing relevant skills in programming, data retrieval from databases and literature, data analysis, and omics data integration. These involve concepts of reproducibility and the Findable, Accessible, Interoperable, and Reusable (FAIR) principles in research. The early exposure of students to these topics is an added value to their education [4]. The reporting and presentation phase of PBL helps the students to further develop these soft skills, which are becoming more and more relevant. Presenting your results is an excellent opportunity to develop the “art” of giving engaging speeches. Independent of achieving the initial project goals or not, an indicator of the success of the PBL approach is the ability of the students to develop active thinking, including pinpointing critical aspects or strengths of the project, as well as the ability to formulate relevant questions, draw biological conclusions, or to foresee possible developments [5]. Interaction, brainstorming, and mentoring by the teacher contribute positively to these learning goals. This nontrivial engagement required during the courses, from both teachers and students, is the key to their success, as it is the leading force in knowledge acquisition [6].

Despite its huge benefits, PBL is not yet embedded routinely in mainstream educational programmes in computational biology. The preparation of the course itself is not an easy task. As computational biology classes are often very heterogeneous, including students with different backgrounds like engineering, biology, and computer sciences, it is often challenging to design a course that keeps the engagement of all students high. PBL, however, is a straightforward way to help them enter such a multidisciplinary field as it allows them to individually tailor the learning process. Working on a project may thereby reinforce previous knowledge, but also expose students to new topics. This encourages collaborations to efficiently tackle the problems at hand. Having that in mind, choosing the right project to work on is particularly relevant for a group, as it should consider the level of all the students, the ability to adapt individual learning styles and pace, and their likelihood to fulfil the requirements of the projects [7].

In this paper, we describe the structure of our PBL-based computational biology course focusing on metabolic network modelling. We also reflect on our experience and possible improvements. This is based on a 4-year experience of running the course ISB705: Advanced Systems Biology II as a PBL course within the Master in Integrated Systems Biology (MISB) at the University of Luxembourg (http://misb.uni.lu). The MISB is a 2-year curriculum that aims at educating a new generation of interdisciplinary scientists who can handle and analyse large biological data sets by applying bioinformatics and network approaches while understanding the underlying biological principles. The 120 ECTS programme, which combines experimental lab and computer science training, accepts up to 18 students with a bachelor’s degree in biosciences, bioinformatics, or related fields. This paper includes a detailed description of the last course edition and the collected statistics from the last 3 years of course (2019, 2020, and 2021). It has been jointly written by teachers and students, and as that, it includes reflections of both.

## Methods

### Course structure and content

The ISB705 course is running as a 2-week full-time course (9 am to 5 pm) and includes lectures, guided practical, and PBL as detailed in the schedule (**Table 1**). Alongside a detailed description of the course content, the **intended learning outcomes (ILOs)** and the respective aligned **teaching and learning activities (TLAs)** are given below.

**Table 1:** Schedule of the 2-week course.

**WEEK 1**	**MONDAY**	**TUESDAY**	**WEDNESDAY**	**THURSDAY**	**FRIDAY**
09:00	**Lecture**	**Practicals**	**Lecture**	**Practicals**	**Independent study time**
10:00	CBM	Introducing COBRA methods with simple CBM models	Large-scale modelling and omics data integration	Large-scale modelling and omics data integration with rFASTCORMICS	
11:00					
12:00					
12:30					
13:00	**Practicals**	**Practicals**	**Practicals**	**Practicals**	**Report writing (Assignment 1)**
14:00	Introducing COBRA methods with simple CBM models	Introducing COBRA methods with simple CBM models	Large-scale modelling and omics data integration with rFASTCORMICS	Large-scale modelling and omics data integration with rFASTCORMICS	
15:00					
16:00				**Project preparation**	
**WEEK 2**	**MONDAY**	**TUESDAY**	**WEDNESDAY**	**THURSDAY**	**FRIDAY**
09:00	**Independent study time**	**Project-based practicals**	**Project-based practicals**	**Project-based practicals**	**Final project presentations (Assignment 3)**
10:00					
11:00					
12:00					
12:30					
13:00	**Project pitches (Assignment 2)**	**Project-based practicals**	**Project-based practicals**	**Project-based practicals**	
14:00					
15:00					
16:00					

Lectures, guided practical, and PBL times are depicted in blue, pink, and green, respectively. There are 3 assignments (in yellow) contributing to the final grading: a report on the guided practical of week 1, due on Monday after the course ending; a project pitch; and a final project presentation, both evaluated jointly by the students and the teachers. Allocated time for the presentations per student (7 to 15 minutes) is adapted according to the overall number of students. Independent study time is planned to allow the students to prepare for the assignments.

CBM, constraint-based modelling; PBL, project-based learning.

### Lectures (week 1)

The ISB705 course includes 2 morning lectures on constraint-based modelling (CBM) during the first week. The first lecture aims to refresh knowledge gained from the preceding ISB701 course: Introduction to Systems Biology, more specifically on Chapter 2: “Metabolic modelling” of the course handout written by T. Sauter and M. Albrecht (publication in preparation), which is provided to each student. During the ISB701 course, students learned the main concepts of systems biology and metabolic modelling applying ordinary differential equations and CBM and how to solve small exercises and problems on a piece of paper. In the ISB705 course, the students have to solve similar problems, but now using computational approaches within MATLAB and the COBRA Toolbox [8]. Specific topics of this lecture thus are linear algebra, network modelling, and MATLAB; COBRA Toolbox within MATLAB; reconstruction of metabolic networks; properties of the stoichiometric matrix; flux balance analysis (FBA); and automated reconstructions. The second lecture treats large-scale modelling and omics data integration into metabolic models and their applications, which is, e.g., identifying and targeting cancer-specific metabolism with a medium size network-based drug target prediction [9]. The PBL concept of the course is also introduced within these lectures.

### Practical—Monday and Tuesday (week 1)

**Fig 1:**
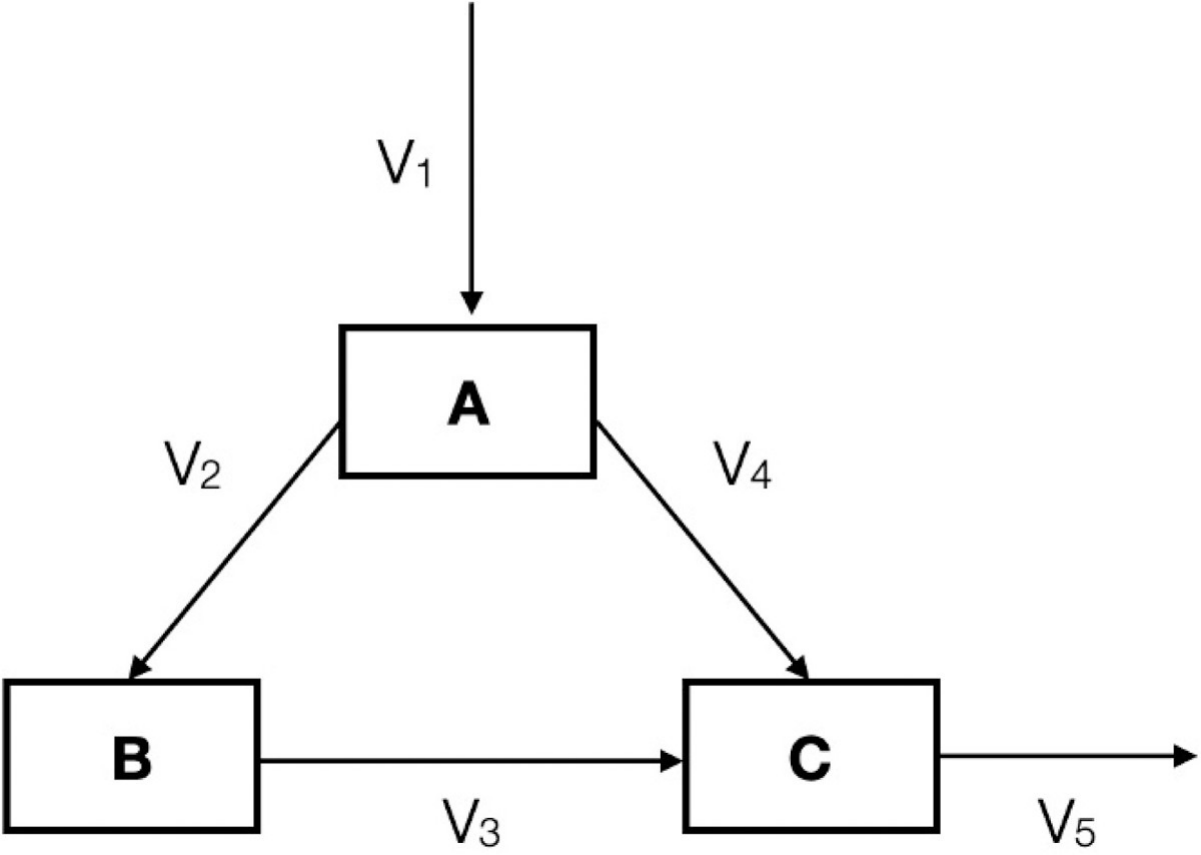
Toy metabolic model analysed during Monday. Metabolites are represented as boxes, and reactions are depicted as arrows. The toy network contains 3 metabolites (A, B, and C) and 5 reactions (v1 to v5).

The first 2 days start with a lecture on the COBRA Toolbox [8] and a tutorial using a small and easy-to-follow toy model (Fig 1). This allows the students to review the core concepts of CBM. Afterwards, a hands-on session on the small model practically shows how to use the functions in the COBRA Toolbox and how to interpret the results of the model analysis.

#### Monday

**ILOs:** Students learn basic usage of the COBRA Toolbox V3 in MATLAB. They get an overview of CBM, including metabolic network reconstruction, FBA, and its applications.

**TLAs:** Hands-on demonstrations and exercises, student–teacher interaction.

Based on a list of biochemical reactions provided by the tutors, the students are asked to create 2 small toy models (example given in Fig 1). The models are small enough (with a maximum of 10 reactions and 6 metabolites) to ensure that students can easily understand and review core concepts of CBM. Afterwards, they answer basic model-related questions, such as “What is the optimal flux distribution for a given objective?” or “Which flux range can this reaction carry?” by using COBRA functions. Among the others, they apply FBA, flux variability analysis (FVA), and random sampling on the models. They compare and discuss the results obtained with each method and observe how the addition of constraints affects the solution space. Further, the students learn how to calculate the maximal growth rate or to perform an in silico single gene deletion study to identify the genes that affect the value of the objective function.

#### Tuesday

**ILOs:** Students learn how the solution space is influenced by adding constraints to the models. They become familiar with COBRA methods on the given medium-sized models.

**TLAs:** Hands-on demonstrations and exercises, student–teacher interaction.

**Fig 2:**
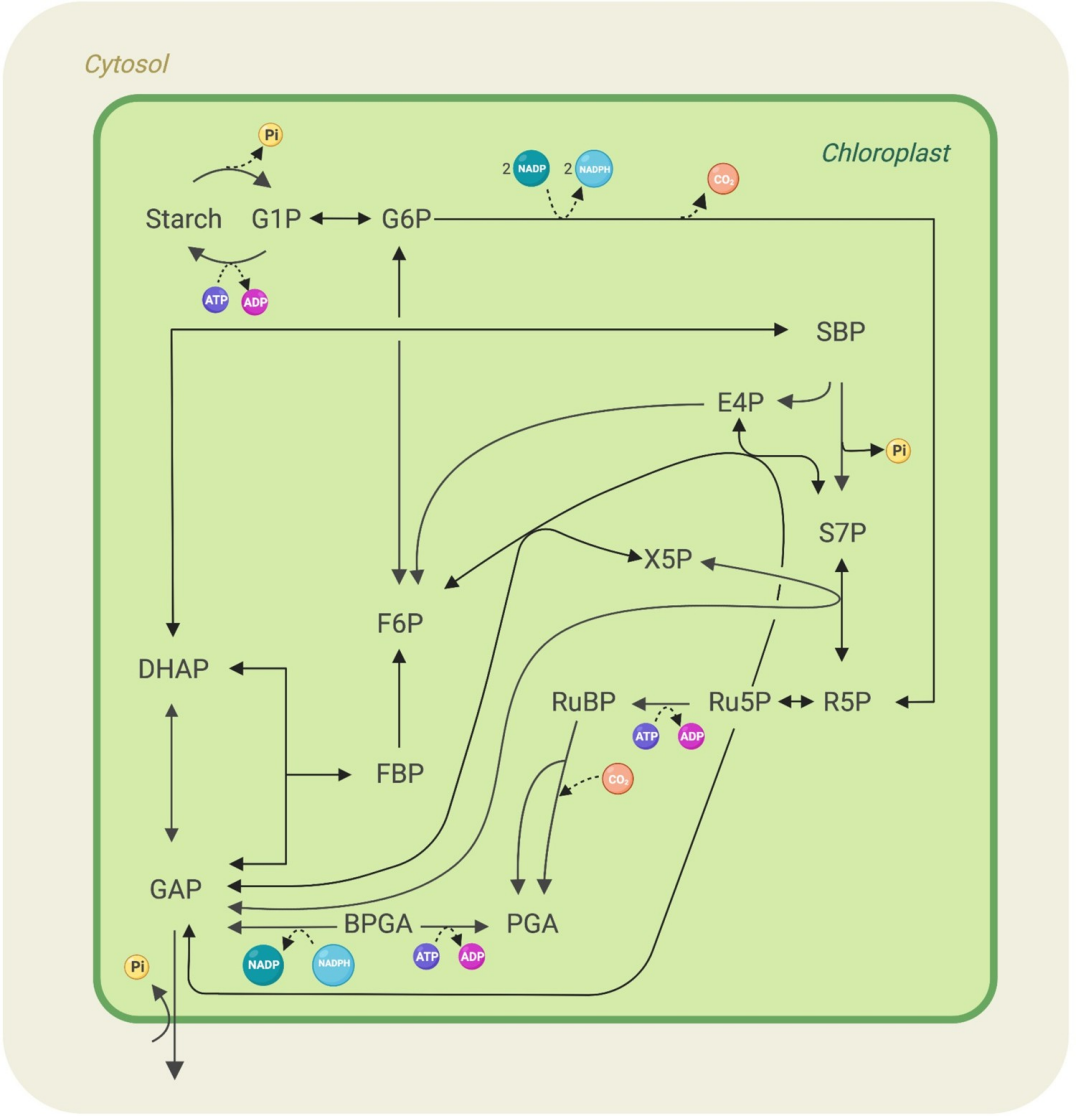
Chloroplast carbon metabolism model analysed on Tuesday. Medium-sized model where metabolites are represented as text and reactions as arrows. The model was adapted from [10].

The students work with medium-sized models such as a chloroplast carbon metabolism model [10] (Fig 2) and an *Escherichia coli* core metabolism model [11], and they are asked to investigate 2 different settings “day versus night” and “normoxia versus anoxia,” respectively, using the first and second model.

First, they investigate whether *E*. *coli* can grow on different carbon substrates in the absence or presence of oxygen and how the growth media and oxygen availability influence the maximum growth rate. Further, by simulating hypoxia, they explore how the growth media affects gene essentiality. Despite the models being still relatively small, these exercises allow a deeper familiarisation with the COBRA Toolbox and the commonly used methods in metabolic modelling.

### Practical—Wednesday and Thursday (week 1)

The third and the fourth day are devoted to the reconstruction and analysis of large context-specific metabolic models using rFASTCORMICS [9] and to tailored drug predictions with the use of an interactive script. After 2 days to earn confidence, a large model is now used as a generic input reconstruction.

#### Wednesday

**ILOs:** Students learn how to extract a context-specific model from a generic reconstruction. They understand the difference between automated reconstructions and manual curations.

**TLAs:** Hands-on demonstrations and exercises, student–teacher interaction.

In the afternoon, students are guided along the model reconstruction process by using the human Recon3D model [12], which contains 10,600 reactions, 5,835 metabolites, and 2,248 genes, whereas the presence of these reactions in specific human cells depends on the tissue, cell type, and other contextual information. Specifically, they get insights into the input model structure, data visualisation and discretisation, building of a consistent input model, medium constraints, context-specific reconstruction, identification of essential genes, and tailored drug prediction. The applied rFASTCORMICS [9] pipeline is described in more detail below.

By using an interactive MATLAB script, during this practical session, each answer is checked automatically, and students receive immediate feedback that allows them to work at their own pace. Additionally, a hint function is included that helps students to find the correct approach and/or function to use to solve a question. Tutors are available for further assistance in the room or online, depending on the setting.

In more detail, the script prompts the students to understand how to handle the structure variable in which the model is stored by facing the given tasks: (i) how to access each field in the structure variable; (ii) how many and what type of elements are stored in each field, e.g., the field *rxns* (for reactions) contains 10,600 elements or entries; (iii) how to determine which reaction belongs to a given pathway (called *subSystem*); (iv) which metabolite is present in which compartment; or (v) which genes control a given reaction.

Further, examples show how the gene–protein–reaction (GPR) rules are encoded in the *model*.*rules* field and how to map expression levels on the reaction depending on the GPR rule.

In the next step, concepts of flux consistency and context-specific models are treated, and the students learn how to obtain a flux consistent network using FAST Consistency Check (FASTCC) algorithm [13] and how to reconstruct a context-specific model using rFASTCORMICS [9].

The script guides the students through the process of candidate prediction for drug repurposing. Here, the students perform in silico single-gene knockouts to predict cancer-specific essential genes and compare the results against *in vitro* CRISPR high-throughput screens. Enrichment tests, such as hypergeometric tests, are performed to assess the quality of the predictions. Finally, the predicted cancer-specific essential genes are used as surrogates for drug targets, and drugs that showed an inhibiting effect on these genes are selected from DrugBank [14] https://www.zotero.org/google-docs/?broken=BGKJfyor another database as potential drug candidates for repurposing.

#### Thursday

**ILOs:** Students get overview of available methods and tools for FBA and metabolic network reconstructions; they learn how to apply the rFASTCORMICS pipeline and how to run other specific tools and to derive biological insight based on the obtained results.

**TLAs:** Hands-on demonstrations and exercises, student–teacher interaction.

As a final real-life application of context-specific model building, a breast cancer metabolic model is reconstructed using The Cancer Genome Atlas (TCGA) data and subsequently analysed. A total of 25 samples each of breast cancer and healthy breast from the TCGA-BRCA data set [15] are randomly selected. Data are obtained in Fragments Per Kilobase of transcript per Million mapped reads (FPKM). The example aims at explaining the rFASTCORMICS pipeline [9] in terms of outlier detection using principal component analysis (PCA), sanity check of the discretised data, comparing the context-specific model, e.g., comparing that pathways have been included/excluded from the reconstructions (pathway presence rate), and performing in silico single gene deletions, each with respective visualisation figures.

### PBL (mainly week 2)

**ILOs:** Students apply CBM techniques learnt in week 1. They develop, run, and present their project idea. They solve problems, interpret the results, critically think, and suggest possible improvements.

**TLAs:** PBL that includes hands-on sessions, literature search, presentations, brainstorming, teacher–student interaction, and student–student interaction.

The PBL concept of the course is introduced on Monday of week 1 within the lecture on CBM and emphasised again on Wednesday. Besides some general statements on PBL, examples of successful pitches and final presentations are shown.

The work in the second week consists of 3 parts, each one is accompanied and supported by tutors.

#### (i) Development and presentation of a CBM project on Monday

All the students are advised to develop their project ideas in the field of metabolic modelling, based on their research interests. This can, e.g., be done by taking a published research paper as a starting point. Hints for model and omics databases are shared. One can find genome-scale reconstructions in repositories such as BiGG Models [16], Virtual Metabolic Human (VMH) database (https://www.vmh.life) [17], and Human Metabolic Atlas [18]. Such generic reconstructions can already be used as models, or a context-specific model can be built from them using preferably the rFASTCORMICS pipeline [9] and RNA sequencing (RNA-seq) data as input retrieved from Gene Expression Omnibus (GEO) [19] or other expression data repositories. The integration of other data types is possible as well. One commonly used strategy is to take a publication that matches the interest of the student to verify if the results in the paper can be reproduced and then to find new applications or questions that were not considered in the original paper.

The research topic, the underlying model(s), and data sets, as well as the formulated research questions, are discussed and checked for feasibility with the tutor. The tutors play a key role in developing a motivating and interesting project and later keep it going by helping to troubleshoot major technical obstacles.

#### (ii) Execution of the project from Tuesday to Thursday

Every single student presents his/her chosen project in a 7- to 15-minute pitch, depending on the overall number of students. Afterwards, everyone is involved in a peer review process where both teachers and students grade each presentation/idea. The students’ average is kept as a grade for the presentation if it is higher and at most 1 point above the teachers’ average (on a scale of 20). At this point, a ranking is made, based on grades, and only top projects are selected for the execution phase. The students are then divided into groups and work as a team for the following 3 days, each group on a selected project. A maximum of 2 projects per available tutor is recommended. On Tuesday, the projects are kicked off by a teaming phase where the teams discuss the details and establish a work plan with the tutors. At least 1 team meeting per day is held during the following 3 project days. Additional consultation is possible on request, but at this stage, the tutors need to find a balance between helping and motivating, but letting the students drive their projects and take ownership. Very often, students work on different tasks in the team, thus using a jigsaw approach that helps reducing individual workload, favours learning, and scales down teachers’ intervention.

#### (iii) Presentation and discussion of the obtained project results on Friday

On the last day, all the students are requested to present the work done in 10- to 15-minute presentations, depending on the group size, followed by a 5-minute Q and A session. Project results (figures) are allowed to be shared during the final presentation, but introduction and discussion must be original for each member of the team.

As most projects will not be completed, a large emphasis can be given to the discussion and outlook. For each presenter, grading is done jointly by students and teachers again.

To further consolidate the achieved learning, students were invited to join the collaborative reflection and writing of this educational paper together with the teachers.

### Assignments

There are 3 assignments in this class that contribute to the final grading of the students:

(i) a report on the guided practical of week 1, in which each exercise of the practical must be explained and completed;

(ii) a short presentation (pitch) describing the project at the start of week 2. The aims, data, models, and workflows to be used in the project must be presented. Students are assessed by their peers and by the tutors; and

(iii) a final project presentation at the end of week 2. The final presentation needs to include the obtained results, a discussion of difficulties encountered during the project, and solution strategies along with an outlook of future work possible or necessary. This presentation is again assessed by both the students and the tutors.

The report, the pitch, and the final presentation account for 50%, 10%, and 40% of the overall grade, respectively, as reported in Table 2. These percentages might be adapted to the actual workload. To grade their fellows, the students are asked to consider the clarity of the presentation, the coherence of the work, as well as the novelty/innovation of the idea, the feasibility of the project/the availability of resources, the topic relevance, and the presentation style.

**Table 2:** Course assignments criteria and evaluation.

Assessment tasks	Type of assessment	Grading scheme	Weight for final grade
Task 1	Take-home assignment	20 points (0 to 20)	50
Objectives	Report on practical tasks of week 1		
Assessment rules	Correctness and discussion of obtained results		
Assessment criteria	Correctness and discussion are equally weighted		
Task 2	Presentation	20 points (0 to 20)	10
Objectives	Present/pitch own project idea		
Assessment rules	Oral presentations at the beginning of project		
Assessment criteria	Quality and style of presentation		
Task 3	Presentation	20 points (0 to 20)	40
Objectives	Present and discuss own project results		
Assessment rules	Oral presentations at the end of project		
Assessment criteria	Quality and style of presentation; Significance of obtained results		
Task 4	Attendance	Pass/fail	
Objectives	Minimal attendance required for this course		
Assessment rules	Minimal attendance required for this course		
Assessment criteria	80%		

### Independent study time

Independent study time was planned in the schedule to allow the students to write their report and perform the literature and data search to define a coherent project.

### Software

Before the course, MATLAB (https://www.mathworks.com/products/matlab.html) is installed on the teaching or students’ laptops along with the “Statistics and Machine Learning Toolbox” and the “Curve Fitting Toolbox” (available from the official MATLAB Add-Ons).

Further required software includes the COBRA Toolbox V3 (https://opencobra.github.io/cobratoolbox/stable) and, optionally, the RAVEN Toolbox (https://github.com/SysBioChalmers/RAVEN), rFASTCORMICS (https://github.com/sysbiolux/rFASTCORMICS) along with the IBM cplex solver (freely available for academics, https://www.ibm.com/products/ilog-cplex-optimization-studio), RStudio (https://www.rstudio.com), and R-cran (https://cran.r-project.org).

The courses in the summer semesters 2020 and 2021 were held in hybrid teaching mode with some students attending on campus and some remotely. Webex 2.0 was used to communicate with the students and tutors on-site and off-site. To facilitate the sharing of scripts and data between students and tutors, each student had a folder on dropit (a folder sharing web application for staff at the University of Luxembourg, available at https://dropit.uni.lu) that was accessible to all participants.

### Prior knowledge

The student should be familiar with basic programming (preferably in MATLAB) and the execution of scripts and functions. A basic understanding of biology and metabolism is also needed.

### Educational staff

We successfully run this course already with up to 20 students and with 3 tutors. We recommend 2 projects per tutor and grouping of students according to the maximal number of students. Thus, for larger classes, the students can be arranged in slightly bigger groups, to not overwhelm the tutors. Also, in larger groups, a jigsaw approach, where each student can run a specific task of the project, can be applied, thus resulting in bigger classes but fewer tutors’ involvement, unless needed.

### Useful resources

Transcriptomic or gene expression data (such as microarray or RNA-seq data) is required for the model reconstruction with rFASTCORMICS [9]. This data can be retrieved from public repositories such as GEO (https://www.ncbi.nlm.nih.gov/geo [19]) or ArrayExpress (https://www.ebi.ac.uk/arrayexpress [20]). Other resources such as cancer patient data available from the TCGA consortium [21] or cancer cell line data from the CCLE [22] database can also be used.

Models were retrieved from databases and websites such as BiGG Models (http://bigg.ucsd.edu [16]), the Human Metabolic Atlas (https://metabolicatlas.org/gems/repository [18]), and the VMH (https://www.vmh.life [17]).

Rcran and RStudio [23] with their numerous open-source packages allow preprocessing and cleaning the data, perform identifier conversion or visualisation of the data.

Toolboxes, such as the COBRA Toolbox [8] and the RAVEN Toolbox [24], contain modelling and analysis scripts in MATLAB and/or in python that can be used. Some in-house scripts were provided by the tutors, i.e., to fix issues with the models or to facilitate the visualisation of the results. Websites such as Human Metabolic Atlas [18] and VMH database [17] facilitate the mapping of identifiers between models and between the model’s components and the data (i.e., medium composition).

Moreover, a detailed tutorial to run rFASTCORMICS [9] and its wiki page are available on our GitHub account: https://github.com/sysbiolux/rFASTCORMICS.

## Results

The individually developed PBL projects covered a wide range of aims, organisms of interest, models, and data sets (**Table 3**) and are united by the application of CBM of metabolic networks. Projects included a variety of different specific methods, like context-specific network reconstruction, optimisation of a given metabolic function, FVA, flux sampling, essentiality analysis applying single and combinatorial gene knockouts, constraining the growth medium, etc. As these methods and the respective results were discussed in the classroom during PBL and within the student groups, as well as during the final presentations, all students got a basic exposure to these techniques. The final presentations also gave an excellent summary of CBM and its possible applications in different fields of research. During the optional drafting of their project summary for this paper, some students condensed the obtained results and lessons learned further (see S1 Appendix), under the continued mentoring of their tutors and the course director.

**Table 3:** Overview of selected student projects in the PBL of week 2 of the 2020 and 2021 course editions.

Project	Aim	Organism(s)	Models (draft/context)	Databases and tools	Outcome(s)
P1: Engineering a synthetic pathway for maleate in *E*. *coli*	Building a model that includes all the necessary reactions for the maleate production and checking which other reactions of the model could be optimised to get a higher maleate production	*E*. *coli*	*iEC1344_C* [25] (BiGG Models)		Creation of an *E*. *coli* that was survivable and able to produce maleate. Identifying other reactions that could be optimised to increase the maleate production
P2: Impact of different diets on anxiety and depression in regards to serotonin levels	Evaluating the growth of 3 *Clostridial* species for holding a high 7α-dehydroxylation activity that strongly up-regulates serotonin production	*Clostridium hiranonis* *Clostridium hylemonae* *Clostridium scindens* *Bacteroides fragilis* *Bacteroides uniformis*	*C*. *hiranonis* TO-931 DSM 13275 *C*. *hylemonae* DSM 15053 *C*. *scindens* ATCC 35704 *B*. *fragilis* NCTC 9343 *B*. *uniformis* ATCC 8492 [26] (VMH models, AGORA reconstructions)		Reduced growth rate of *C*. *hiranonis* growth under an unhealthy diet, which might affect serotonin levels
P3: Genome-scale metabolic modelling of human CD4+ T cells	Building models for naive CD4+ T cells, Th1, and Th2 cells and performing a comparative essentiality analysis	*H. sapiens*	Recon3D	GSE22886 [27], GSE24634 [28], and GSE2770 [29]	Identified essential metabolic pathways for naive CD4+ T cells, Th1, and Th2 cells
P4: Exploratory study using a human alveolar macrophage or respective mouse model combined with the Zika virus	Performing single reaction deletion, after the reconstruction of the host virus model, to predict putative antiviral strategies that could be tested experimentally	*H*. *sapiens* *M. musculus* Zika virus	Human macrophage *iAB-AMØ-1410* [30] Mouse model *iMM1415* [31] Zika virus Biomass objective function [32]	DrugBank	Confirmation of the validity of the human virus model and reconstruction of other models using different hosts Identified potential targets for antiviral therapies
P5: Metabolic differences in high and low STAT3 expressing breast cancer	Comparison of metabolic reactions, genes, and metabolites in high and low STAT3 expression using expression data from breast cancer patients and a breast cancer cell line model	*H*. *sapiens*	*MDA-MB-231*: Context-specific breast cancer cell line models [33]	GSE62944 [15]	Phenylalanine and vitamin B6 metabolisms identified as unique in the low STAT3 expression model

Project title, aims, organisms of interest, used models, and data, as well as project outcomes, are briefly summarised. More detailed descriptions of the projects are given in S1 Appendix. These descriptions have been written by the individual student as part of the learning process.

PBL, project-based learning.

In concordance with the educational aims of PBL, a variety of learning outcomes were achieved with the course. Based on a concise introduction of the core concepts of methods of CBM in week 1, students managed to develop and execute their real-life research problems in week 2. In close interaction with tutors, they managed to pick up and apply additional CBM methods and presented these and the respective results to their peers. Students moved forward at very different speeds. Learning took place in a tailored and personalised manner. Not all initially planned aims could be reached, partially due to overambitious plans and also unforeseen technical issues. But the discussion of these issues supported by the tutors allowed the students to understand the observed problems and present a meaningful outlook on how to continue the project if time would allow. During the project work, the involvement of the tutors was usually high, and a sense of team spirit emerged within the student group, but also with the tutors. An active communication was key and was initiated first by the tutors, but then more and more by the students. Many students were very engaged and moved from being overwhelmed to a phase of being passionate and taking ownership in their projects. This culminated in mostly very good and engaging final project presentations.

**Fig 3:**
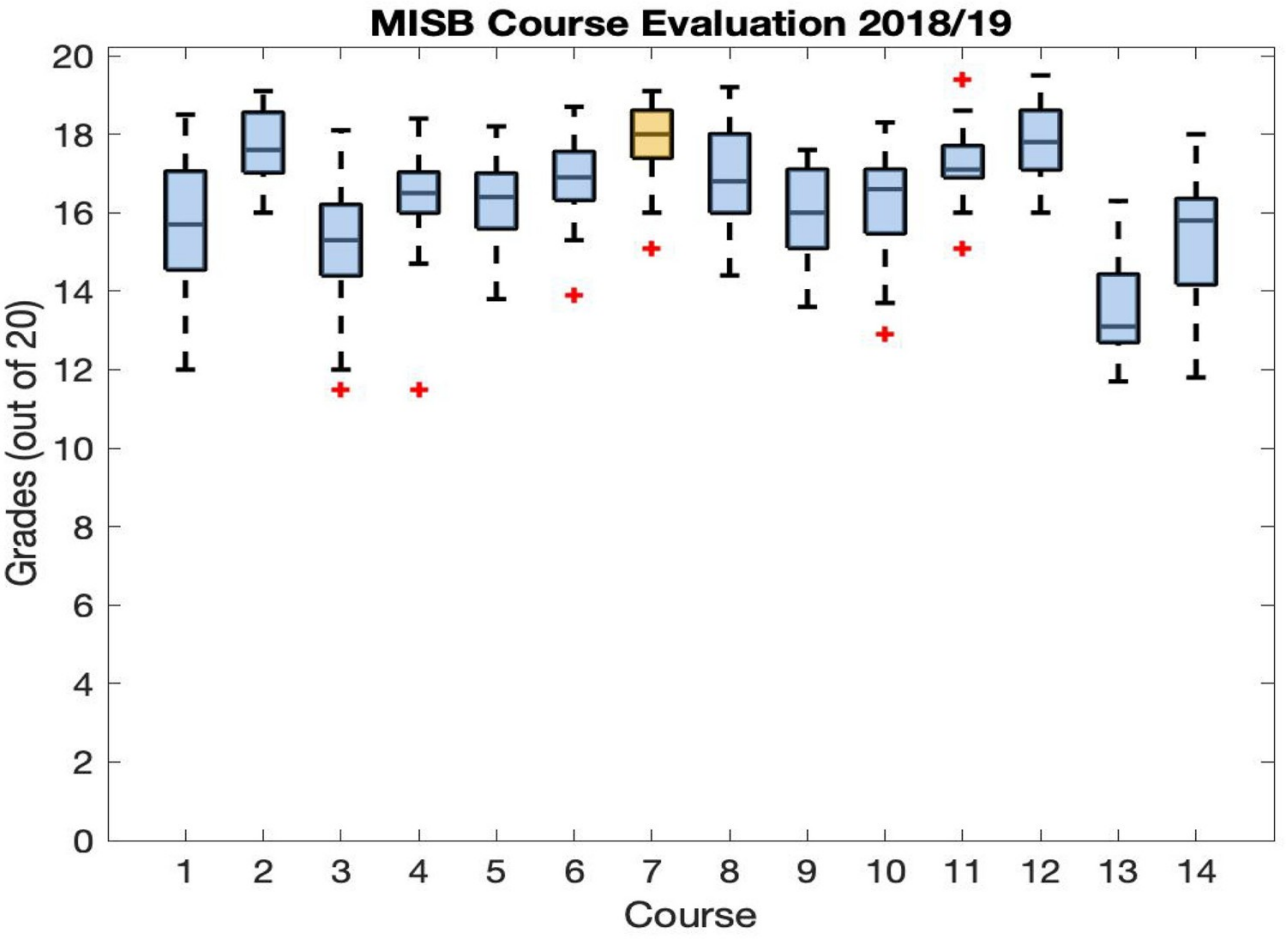
Student’s course evaluation scores for the academic year 2018 to 2019. The Advanced Systems Biology II course (number 7, in yellow) has been the most appreciated by the student’s population. MISB, Master in Integrated Systems Biology.

The overall successful execution of the course is also reflected by the evaluation that the students made. In the academic year 2018 to 2019, it was the most appreciated by students, as it obtained the highest score compared to the other courses in the master (Fig 3). This statistic is based on Table 4, which contains the questions that the students answer for the evaluation of all courses, and it embeds the scores they gave to the Advanced Systems Biology II course, edition 2018 to 2019. Overall, this anonymous course evaluation gave mostly very good to good ratings. The respective detailed comments of the students are included in the following discussion jointly collected by teachers and students. Already from these comments and the table, it is possible to see that the students really appreciated the teaching methods used in the course, the competence and motivation of the teaching staff, as well as the interaction between the parties.

**Table 4:** Evaluation form and results of Advanced Systems Biology II course, edition 2018 to 2019.

ISB705: Advanced Systems Biology II Evaluation Form 2018 to 2019						
	+++	++	+	-	--	---
I spent enough time studying to attain the level of deep learning	6	7	1			
In this study module, the students were encouraged to think critically	8	6				
There was an encouraging and motivating atmosphere in the study module	9	5				
I was active during the study module	11	3				
I was motivated to learn	7	6	1			
I learned new things during the study module	9	5				
I understand why the things to be learned in the study module were important	7	6	1			
I was able to make connections between new learning and prior learning	6	6	2			
My understanding of the subject matter increased during this study module	9	5				
I received enough guidance during the study module	10	4				
The teacher was in my opinion motivated to teach	11	3				
In my opinion, there was enough interaction during this study module	11	3				
In this study module, methods that activated my thinking were used	6	7	1			
The teaching methods used enhanced my learning	2	11		1		
I found the learning material for this study module quite easily	5	5	3	1		
In my opinion, the learning materials were up to date	7	7				
The study module assessment was fair in my opinion	5	8	1			
I knew what the assessment criteria were during the study module	9	4	1			
The learning arrangements in this study module worked well	7	7				
The teacher was competent	11	3				
The teacher was able to concentrate on the most relevant matters	9	5				
The study module was too demanding for me	2	3	7	1	1	
The study module progressed logically and coherently in my opinion	7	5	2			
The practical/internship training helped me to understand the meaning of prior theoretical learning	7	5				
I planned my time when studying this study module	4	8	1	1		
I spent enough time studying in this study module (1 credit = 25 hours of work)	7	6	1			
In my opinion, the study module load corresponded with the credits given	6	7	1			
I was able to manage time during the study module	3	6	4	1		

**Fig 4:**
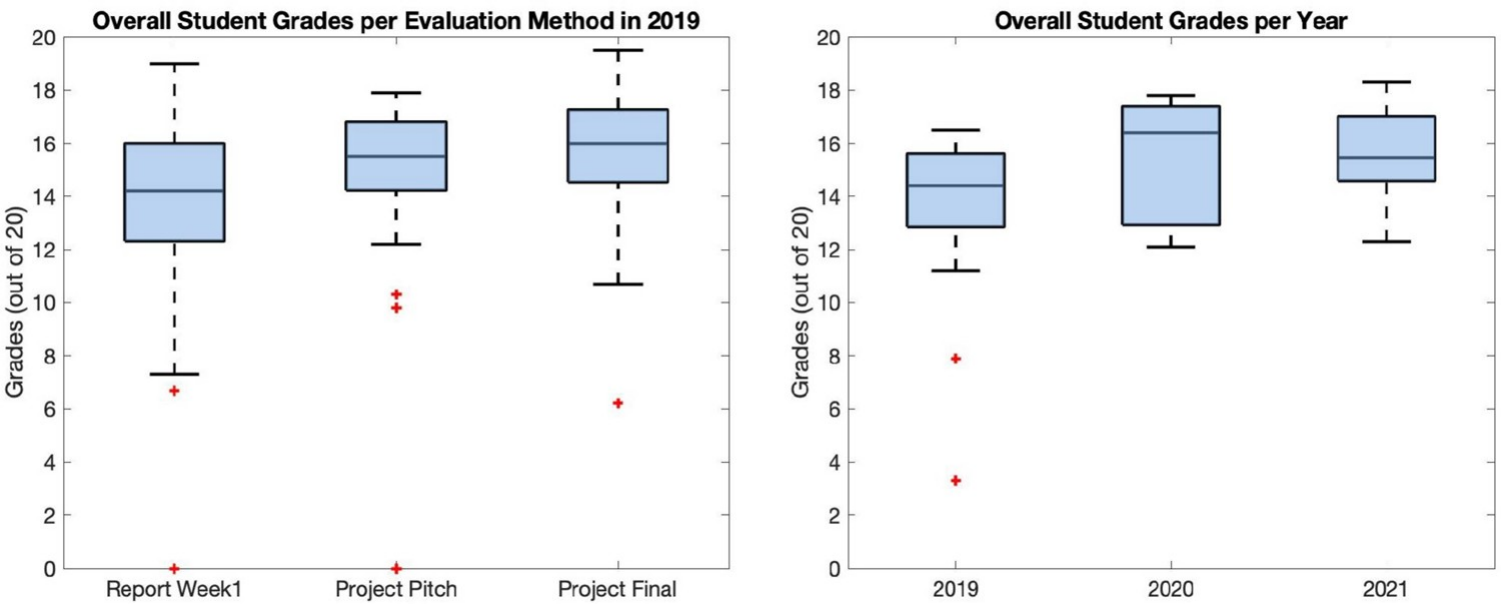
**(A)** Overall student’s grades per evaluation method: The highest scores are obtained by the students in the final project, meaning that the PBL approach has a good impact on their performances. **(B)** Comparison of students’ grades per course edition: The graph shows a year-by-year improvement in students’ performances, which, in our opinion, also stands for the quality of the teaching improvement, including the course’s methods and contents. PBL, project-based learning.

Interestingly, the grades for the reports on the guided practical of week 1 were a bit weaker (fair / (very) good / good) compared to the grades of the project pitches (good / very good / (very) good) and the final project presentations ((very) good / very good / very good-excellent) as shown in Fig 4A, supporting the idea that PBL positively impacts their performances and that the TLA adopted was very tailored to the ILOs planned.

Finally, assessing the underlying numerical grades of 20 to 0 (with 20 being the top grade) on a qualitative scale of excellent / very good / good / fair / passed / not passed (with not passed being below 10/20), the average overall grade for the last 3 course editions were good / (very) good / very good, which largely stands for the success of the course.

Moreover, the comparison of students’ grades for the same period is also presented in Fig 4B, witnessing that students’ performances have improved year by year, which also reflects teaching/course quality improvements.

## Discussion

### Lessons learned: What worked?

The students came up with very different and interesting project ideas that could, in the long run and for some of them, be turned into master thesis projects. To come up with feasible ideas, they first had to gain a good understanding of CBM in terms of what can be modelled and what would be required for the modelling. The difference to other modelling approaches like kinetic modelling was becoming clear. Students needed to search for a model and the respective data on their own and had to decide which method learned in week 1 could be applied to achieve specific aims and if these methods are readily available, i.e., in the COBRA or Raven Toolboxes.

The students had to actively confront themselves with questions that regularly come up when designing a project and had to find suitable answers with the help of the tutors. This concerned, e.g., the availability and quality of resources, as well as timing and sharing of the different work packages. In a more classical practical course, students would usually follow a well-defined and optimised script and project plan that would not leave much room for errors and would not necessarily initiate deeper thinking about the different steps. Errors allow pinpointing the not fully understood concepts and limitations of the methods that could easily have been overlooked in the amount of information of a 2-week course. Students got hands-on experience on CBM and learned how to solve technical or scientific problems arising in their project. Involvement, active thinking, a positive atmosphere, competent teaching staff, and enough guidance are what students particularly appreciated, as emerge from the course assessment of 2019. On the other hand, the high involvement was a double-edged sword for some students, who also declared that the availability of models and data needed during the project phase, as well as the time constraints, is what made the course highly demanding in their opinion (specific comments can be found in Table 4).

Finally, the students had to present their project twice, once at the beginning as pitch and once at the end showing and discussing the obtained project results. Thereby, they could critically assess the feasibility of the initially planned work and the validity of assumptions and results of the research paper underlying their project. The interpretation of the obtained results was quite interesting as the students often proved a critical mind towards their work but also towards the paper, data, or model used as a backbone for their project. They better understood that published data or models are not always of good quality. Their project thus showed them the importance of quality control and critical thinking in general.

### Lessons learned: What is challenging, and what could be strengthened?

The main challenge of this course is what makes it interesting. Students can come up with very different ideas including a multitude of data types, models, organisms, and analysis pipelines. Therefore, the first challenge can be the required data preprocessing. The data are not always in a usable format and has to be converted, e.g., from counts to FPKMs for the rFASTCOMRICS pipeline, or might be of poor quality. It would be useful if students already had a concrete idea on the project, the respective needed data, and models at the end of week 1. This would allow the tutors to check the quality of the inputs and, if necessary, prepare some preprocessing to allow the students to put more focus on the modelling and to avoid changing the data set during the project.

Structuring the projects as a hackathon where students would have one goal to achieve (e.g., identifying known metabolic essential genes from previously determined expression data) might be helpful. Moreover, it may help reduce the workload for teachers in the practical and allow for better teamwork of the students. On the other hand, it may limit the creativity of the students in the case of previously determined data and known solutions. Moreover, a hackathon may provide an opportunity to crowdsource the students’ efforts for solving problems using metabolic modelling where students would be free exploring different pipelines.

Besides the challenges related to the diversity of topics, there is also the heterogeneity of students’ backgrounds that needs to be taken into consideration. Some students have very limited programming skills, whereas others had already worked with COBRA models in the past (e.g., during their previous studies). Thus, a better learning outcome may be achieved by integrated blended learning concepts, e.g., by asking the students to watch prerecorded lessons and fill respective assignments. This would help in kick-starting the basic prerequisites for the course, such as introductory level in MATLAB and basic bioinformatics skills in exploring GEO, etc. Moreover, this could be an easy solution to have the course for larger classes, as it would potentially reduce the workload for teachers.

The Coronavirus Disease 2019 (COVID-19) pandemic made interactions with the students more difficult, despite the technical solutions for hybrid teaching. Guiding students remotely on such a project is difficult as the tutors need to spend time with the individual students to check their code, discuss the details of the projects, and clarify certain doubts. When the lectures were performed remotely (in the summer semester 2020), this was done in one-to-one calls. Especially towards the end of week 2, students sometimes had to wait until tutors were available, which could be frustrating if just a quick answer on a detail is needed, despite the available Q and A forum on the Moodle learning platform. Further, the positive effect of learning by eavesdropping from other discussions in the same physical classroom is decreased in an online setting. Having a hybrid teaching setting in the summer semester of 2021 made tutoring and communication somewhat easier, as only few students were attending the course remotely and could work in a small group with a remote tutor. Nevertheless, for the hybrid format, dealing with lectures on-site and online at the same time is quite difficult as one needs to constantly make sure that the online students can see the board or that students online can hear questions asked by the audience. The sound of the class or from Webex had to be muted to allow working on the small groups and to reduce distraction from discussions on other projects. This created 2 separated groups, with the remote tutors and students not necessarily receiving all instructions and information.

Finally, concerning course assignments and technical issues, having reports as assignments for the guided practical of week 1 is not optimal as reports tend to be passed along from one generation to the other, forcing the tutors to renew and adapt the tasks regularly. Additionally, there have been some unforeseen minor issues with the interactive MATLAB script (Wednesday and Thursday of week 1). For example, it was not tested on a machine running macOS beforehand, which caused some problems later as some students used their private Mac machines. Furthermore, each machine runs slightly differently, which can cause slightly different results due to numerical issues, and some preset answers had to be changed to adapt to the variety of obtained results. As students were allowed to use their computer for their projects, this can sometimes be challenging for the tutors as students might have different operating systems, have different versions of the used software, or not have all the toolbox installed or working because some dependency is outdated. A common source of problems was, e.g., an outdated version of Java. Hence, we are exploring new solutions, e.g., creating a virtual machine with all required tools installed that students could simply log in and use.

## Conclusions

This course with guided practical combined with PBL is an excellent way of teaching metabolic network modelling within a Systems Biology or Computational Biology curriculum. It allows the students to work on real-life scenarios and enhances learning. Such a course needs to be prepared carefully and adapted to the exact course content and number of students and available tutors. It requires and facilitates active engagement of teachers and students and is overall a very rewarding teaching experience.

## Supporting information

S1 Appendix Descriptions of selected student projects in the PBL of week 2 of the 2020 and 2021 course editions. These descriptions have been written by the individual student as part of the learning process. PBL, project-based learning.
(PDF)
