# Supplementary material for: Project-based learning course on metabolic network modelling in computational systems biology
Source: PLoS Comput Biol. 2022 Jan 27;18(1):e1009711. doi: 10.1371/journal.pcbi.1009711 (PMC8794106; doi:10.1371/journal.pcbi.1009711)
Supplement: S1 Appendix — These descriptions have been written by the individual student as part of the learning process. PBL, project-based learning. (PDF) [file pcbi.1009711.s001.pdf]

S1 APPENDIX:

Detailed student project descriptions as listed in **Table 3**. Descriptions were drafted by each student individually as part of the learning process at the end of the project work and were only marginally edited by the tutors to leave an impression on the level and diversity.

P1: Engineering a synthetic pathway for maleate in *Escherichia coli* – Tessy Prohaska

Introduction

In science and research, bacteria make a useful and powerful tool for genetic research since, compared to eukaryotes, the size of their genome is relatively small which makes it easier to keep a good overview of the studied organism. One of the most commonly studied bacteria is *Escherichia coli* (*E. coli*) which has a genome size of around 4000 genes whereby the size of the human genome lies around 30000 genes. It is one of the best-studied organisms on earth since it has well-characterized biochemistry and physiology. *E. coli* is used in biomedical research since it is much easier to manipulate its genome without deranging the natural genome and its functions (1). It has become a popular candidate for metabolic engineering to produce chemicals, fuels, pharmaceuticals, and medicine. Maleate is an important dicarboxylic acid which is an essential component used for the synthesis of polymer materials such as pharmaceuticals. In 2017, a research team first successfully modified the genome of *E. coli* by introducing a new synthetic pathway for maleate production (2).

Aim

The project aimed to reproduce the results of the paper published in 2017 by Noda et al which engineered a synthetic pathway for maleate (2). The idea is to choose an *E. coli* model and introduce the needed reactions to recreate the metabolic remodelled *E. coli* model. After adding the new synthetic pathway, the next step would be how far one can optimize certain pathways to increase the maleate production without killing the bacteria.

Material and Methods

The first step was to choose an *E. coli* model for the project. The name of the used *E. coli* model is *iEC1344_C* which can be downloaded from the BIGG Model website. After choosing the model, the next step was adding all the needed reactions to create the modified *E. coli*. The idea of the article was to extend the chorismate pathway so that in the end the *E. coli* can produce maleate by itself. To add the reactions correctly to the model, the KEGG database was used. Eight reactions needed to be added in order that the *E. coli* was able to produce maleate. After creating the modified *E. coli* model, the next step was to see how far one can optimize the maleate production. During this process, biomass production was simultaneously observed, since if one wants to genetically modify an organism to produce a new molecule the newly added reactions mustn't be reducing the life capacity of the organism. The organism should produce as much maleate as possible, but also still produce a certain amount of

biomass. The final research point was to see if one optimizes certain exchange reactions and if this would influence the maleate production.

**Components**	**Counts (Original Model)**	**Counts (Modified Model)**
**Genes**	1344	1349
**Metabolites**	1934	1941
**Reactions**	2726	2734

S1 Table 1. Number of genes, metabolites and reactions of the *E. coli* model before and after adding the needed reactions.

Results

The first step was to create a model which would be survivable in real life. After adding all the new reactions to the original *E. coli* model, the first problem which needed to be solved was that one could not simply push all the fluxes in the direction of maleate since then the biomass production would be zero which means that the model is not survivable. This problem could be solved by two solutions: optimise for the maleate and biomass production or fix the lower limit of the biomass. The first solution was giving a high biomass production but a rather moderate production of maleate which would not accomplish the goal of this project. The second solution was giving a high maleate production and the biomass production was still high enough which is why this solution was applied. The next step after having created the genetically modified *E. coli* model was to find further reactions which could be optimised to increase the maleate production. The first factor which would increase the maleate production would be oxygen. Increasing the oxygen supply in their living environment or overexpressing all the reactions which produce oxygen as a by-product or main product would increase the maleate production. Adding more minerals such as potassium and calcium in the medium would increase biomass production which would be helpful for the longer survival of *E. coli* but would not affect the maleate production directly. The most interesting finding was that optimizing the exchange reaction of phosphate (uptake) increased the biomass and the maleate production simultaneously.

Summary

During this project, one could see that, theoretically, it would be possible to modify the genome of the *E. coli* so that the organism would be able to produce the essential molecule maleate without perturbing the functioning of the organism. The metabolic modelling showed that the organism can produce enough maleate and still be able to have enough energy left for the life-important biomass reactions. Additionally, further reactions were found which could be optimized by improving the growth and living environment of the *E. coli* like keeping the bacteria in an aerobic environment or by adding feeding supplements such as potassium or phosphorus into the medium of the bacteria. In summary, creating a modified metabolic model

helps in reproducing genetically modified organisms which already gives an idea about the outcome of the afterwards performed experiments.

References

1. Monk, J. M. *et al.* Multi-omics Quantification of Species Variation of Escherichia coli Links Molecular Features with Strain Phenotypes. *Cell Syst* 3, 238-251.e12 (2016)
2. Noda, S., Shirai, T., Mori, Y., Oyama, S. & Kondo, A. Engineering a synthetic pathway for maleate in Escherichia coli. *Nature Communications* 8, 1153 (2017).

P2: Impact of different diets on anxiety and depression in regards to serotonin levels - Daniel Guignard

Introduction

Depression and anxiety are widespread related conditions strongly impacting societies and families with an estimated economic cost of about €170 billion per year in the EU (WHO) (1). Although being multifactorial complex disorders, reduced level serotonin was identified as inducing a causative relationship (2). Surprisingly, this molecule is mainly produced by enterochromaffin cells (ECCs) located in the colon, therefore raising the question of the role played by the microbiota in this condition. Yano *et al.* showed that indeed, some metabolites excreted from bacteria could strongly stimulate the serotonin production of the ECCs (3). Moreover, they could correlate elevated serotonin levels in mice to the increased gut colonization by *Clostridium* species, particularly the ones expressing a high 7α-dehydroxylation activity. Specific models for those bacteria (*C. scindens, C. hiranonis, C. hylemonae*) were retrieved from VMH AGORA reconstructions (model names in **Table 3** in the main text) and their growth was assessed under 3 different diets (Unhealthy, DACH, Mediterranean) from the same database. An unhealthy diet containing a high kcal amount, saturated fatty acids, and cholesterol. The DACH (abbreviated from the international codes of Germany, Austria and Switzerland) being the ideal diet established by the society for Nutrition in Germany, Switzerland, and Austria (4). Finally, the Mediterranean diet is known to be one of the healthiest diets regularly consumed by millions of people mostly containing fresh plant foods, poultry, and olive oil as a fat source (5).

Aim

The aim was to evaluate the growth of the 3 Clostridial species revealed by Yano *et al.* for holding a high 7α-dehydroxylation activity implicated in the synthesis of deoxycholate from cholate. Both molecules strongly up-regulate serotonin production in ECCs. The fluxes going towards the biomass were assessed under 3 different diets retrieved from VMH with specific values for each exchange reaction of the models.

Results

The Clostridial species were assessed together with 2 Bacteroides species, which showed no impact on serotonin level according to Yano *et al.* mice study.

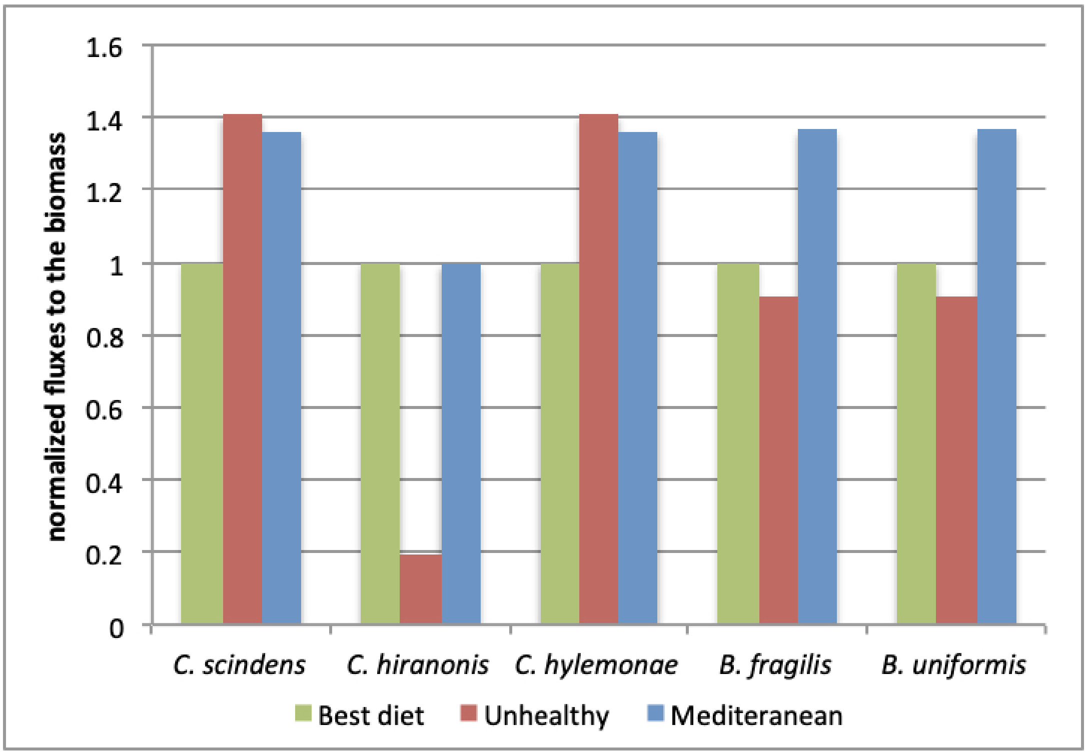

S1 Fig 1. Normalized fluxes on the DACH diet towards the biomass for 3 Clostridial and 2 Bacteroides species.

C. hiranonis growth is reduced under an unhealthy diet. Surprisingly the biomass for the 2 other Clostridial species is increased under the same diet. Another observation is that the Mediterranean diet seems to promote the growth of almost all the Clostridial bacteria, better than the ideal DACH diet. Further investigations should be conducted on the metabolites stimulating serotonin production on ECCs, namely cholate, deoxycholate, and tyramine (3). A constrained-based model for the ECCs using rFASTCORMICS could assess those metabolites on the cellular serotonin production.

References

1. « Depression in Europe: facts and figures». https://www.euro.who.int/en/health-topics/noncommunicable-diseases/mental-health/news/news/2012/10/depression-in-europe/depression-in-europe-facts-and-figures (viewed on April 13, 2021).
2. P. R. Albert, F. Vahid-Ansari, et C. Luckhart, « Serotonin-prefrontal cortical circuitry in anxiety and depression phenotypes: pivotal role of pre- and post-synaptic 5-HT1A receptor expression », *Front Behav Neurosci*, vol. 8, juin 2014, doi: 10.3389/fnbeh.2014.00199.
3. J. M. Yano *et al.*, « Indigenous bacteria from the gut microbiota regulate host serotonin biosynthesis », *Cell*, vol. 161, nº 2, p. 264-276, avr. 2015, doi: 10.1016/j.cell.2015.02.047.
4. I. Elmadfa et C. Leitzmann, *Ernährung des Menschen*. Ulmer, 2004.
5. W. C. Willett *et al.*, « Mediterranean diet pyramid: a cultural model for healthy eating », *Am J Clin Nutr*, vol. 61, nº 6 Suppl, p. 1402S-1406S, juin 1995, DOI: 10.1093/ajcn/61.6.1402S.

P3: Genome-scale metabolic modelling of human CD4+ T cells - Ni Zeng

Introduction

CD4+ T lymphocytes play a crucial role in the adaptive immune system. Depending on the extrinsic and intrinsic factors, naïve CD4 T cells can be differentiated into various subsets of effector T cells, such as Th1, Th2, Th17, inducible Tregs, and so on, to function differently against various pathogens. Metabolism is one of the major factors which profoundly influences T-cell differentiation and function. Distinct T-cell lineages utilize different metabolic programs to fulfil their functions (1,2). Therefore, modulation of CD4+ T-cells metabolic pathways can be useful to fight against some immune-associated diseases. Computational metabolic modelling of different subsets of CD4+ T cells might be a powerful approach to discover new mechanisms, biomarkers, and drug targets for CD4+ T cell-mediated immune diseases.

Aim

The project aims to construct the genome-scale metabolic models of naïve $CD4^+$ T cells, Th1, Th2 cells. Based on the modelling, we aim to find out the metabolic pathways and metabolic genes which are exclusively essential to these three subsets of T cells.

Materials and methods

Transcriptome data collection and processing: Microarray data of human naïve CD4+ T cells, Th1 and Th2 were selected and collected from the Gene Expression Omnibus (3) database. For naïve CD4+ T cells (unpolarized), 9 datasets were included. For Th1 cells, which were differentiated from naïve CD4+ T cells in the presence of IL12, IFNγ, anti-CD3, anti-CD28, and anti-IL-4 antibodies, 11 datasets were collected. For Th2 cells, which were differentiated in the presence of IL-4, anti-CD3, anti-CD28, and anti-IFNγ antibodies, 12 datasets were used (see **Table 3** in the main text). All samples were analysed with (HG-U133A) Affymetrix Human Genome U133A Array. The 32 microarray CEL files were read by the package of BiocManager (1.30.10) in R studio version 1.4.1106 with the *oligo* package (1.54.1). Data were normalized using the *fRMA* package (1.42.0) and the *hgu133afrmavecs* vector. Then the data were discretized with Barcode.

Reconstruction of cell type-specific genome-scale metabolic model: The data from Barcode were loaded into the FASTCORMICS workflow. Genes with a z-score above 3 are considered as expressed genes and define the discretization score as 1. The ones with a z-score below 0 are defined as unexpressed genes and defined as -1. z-score which is between 0 and 3 is discretized as 0, which represents an undetermined gene set. Then the discretization score 1 is mapped to the consistent version of Recon3D via the Gene Protein Reactions Rules (GPR) to generate the core reactions. The biomass objective function from the Recon3D template was used here.

Model validation: Based on the literature that glucose and glutamine are crucial for the proliferation of $CD4^+$ T cells (4,5), Flux Balance Analysis (FBA) from the COBRA Toolbox was performed using different flux rates of glucose and glutamine reactions and then the effect of these flux rates on the growth rate was evaluated.

Essential genes prediction and pathway enrichment analysis: Single gene deletion was performed to predict the essential genes which are related to the growth rate of three subsets of CD4+ T cells. A Venn diagram was generated to find out the essential genes which are exclusive for naïve CD4+ T cells, Th1, and Th2 cells. Then according to the exclusive essential genes, biological processed enrichment analysis was performed via referring to the database of KEGG.

Results

	Naïve	Th1	Th2
**Genes**	1029	945	951
**Reactions**	1538	1579	1614
**Metabolites**	1379	1307	1343

S1 Table 2. Number of genes, reactions, and metabolites in the FASTCORMICS models.

Common essential genes among Th1, and Th2 & naive CD4 T cells	Unique essential genes to naive CD4 T cells	Unique essential genes to Th2 cells
**55 genes were essential in the 3 cell types. No essential gene was unique to the Th1 cell model.**	**SLC16A10, CPT2, ANPEP, SLCO1B1, SLCO1A2, MTAP**	**SLC25A6, PKM**
Cardiac muscle contraction	Bile secretion	Pyruvate metabolism
Oxidative phosphorylation	Renin-angiotensin system	Type II diabetes mellitus
Parkinson disease	Fatty acid degradation	Central carbon metabolism in cancer
Non-alcoholic fatty liver disease (NAFLD)	Cysteine and methionine metabolism	Glycolysis / Gluconeogenesis
Alzheimer disease	Glutathione metabolism	Glucagon signalling pathway
Huntington disease	PPAR signalling pathway	Purine metabolism
Thermogenesis	Protein digestion and absorption	Parkinson disease
Pyrimidine metabolism	Hematopoietic cell lineage	Cellular senescence
Sphingolipid metabolism	Thyroid hormone signalling pathway	Necroptosis
Glycerophospholipid metabolism	Thermogenesis	cGMP-PKG signalling pathway

S1 Table 3. KEGG pathway enrichment analysis for shared genes in the three cell types, unique genes to naive CD4 T cells, & unique genes to Th2 cells. Th1 cells didn't show any unique essential genes compared to naïve and Th2 cells. The top 10 pathways in each list were only selected by p-value.

In our metabolic models for naïve, Th1, and Th2 cells, we predicted 6 metabolic genes which might be exclusively essential for naïve cell proliferation, and 2 exclusively essential for Th2 cell proliferation. Cysteine and methionine metabolism and glutathione metabolism were indicated to be important for naïve CD4 T cells and pyruvate metabolism and glycolysis important for Th2 cells in the enrichment analysis.

Reference

1. Buck, Michael D., David O’sullivan, and Erika L. Pearce. 2015. “T Cell Metabolism Drives Immunity.” Journal of Experimental Medicine 212(9):1345–60.
2. Buck, Michael D., Ryan T. Sowell, Susan M. Kaech, and Erika L. Pearce. 2017. “Metabolic Instruction of Immunity.” Cell 169(4):570–86.
3. Macintyre, Andrew N., Valerie A. Gerriets, Amanda G. Nichols, Ryan D. Michalek, Michael C. Rudolph, Divino Deoliveira, Steven M. Anderson, E. Dale Abel, Benny J. Chen, and Laura P. Hale. 2014. “The Glucose Transporter Glut1 Is Selectively Essential for CD4 T Cell Activation and Effector Function.” Cell Metabolism 20(1):61–72.
4. Patel, Chirag H., Robert D. Leone, Maureen R. Horton, and Jonathan D. Powell. 2019. “Targeting Metabolism to Regulate Immune Responses in Autoimmunity and Cancer.” Nature Reviews Drug Discovery 18(9):669–88.

P4: Exploratory study using a human alveolar macrophage or respective mouse model combined with the Zika virus - Claudia Cipriani

Introduction

Zika virus (ZIKV) is a member of the *Flaviviridae* family transmitted by insect bites. It was isolated for the first time from a rhesus monkey in 1947 (1). The first recorded human infected case was in Uganda in 1952. In February 2016, the outbreak of the ZIKV was declared as a Public Health Emergency of International Concern by the World Health Organization (2). The virus, primarily spread by Aedes mosquitoes, can then be transmitted to humans through sexual contacts, blood transfusions, or vertical transmission from mother to foetus (3). Symptoms caused by ZIV include microcephaly and neuroinflammatory syndrome (4). Because of the absence of approved treatments for ZIKV infection, new therapeutics are needed. In 2018, a genome-scale metabolic model that combines human macrophage cell metabolism and virus biochemical demand was developed (5).

Aim

The project aimed to confirm the validity of a previously published human-virus metabolic model (5). By performing *in silico* reaction deletions, on the viral-infected human model, we can predict possible antiviral drugs. The building of additional models using other tissues or hosts, including the viral biomass, might help to understand the different modes of virulence and to define tissue-specific drugs.

Material and Method

The human macrophage *iAB-AMØ- 1410* metabolic model, was downloaded from *Aller et al*. (5). This model was reconstructed in a previous study (6) based on Recon1 (7), a generic human metabolic model, and transcriptomic data of alveolar macrophages from different patients. This model did not have genes and subsystem compartments assigned. The genome for ZIKV was obtained from the NCBI database with accession number *NC_012532.1*. Then, the authors constructed the final viral biomass objective function with its necessary components attached in the supplementary materials of the paper. The mouse model *iMM1415* was obtained from the paper (8).

Results

The viral biomass reaction was added to the macrophage model. Optimization of the model was performed once for the viral biomass and once for the macrophage biomass, followed by a single reaction deletion where essential genes for the viral biomass and macrophage maintenance were identified. The result for the viral biomass optimization showed the production capacity of the virus, without any concurrent macrophage biomass production. However, this scenario is biologically not realistic, as the host cell needs to preserve its biochemical demands e.g., due to turn-over. To overcome this problem, we set the optimization weights for the macrophage & the virus to 100 & 1 respectively. Also, the upper bound for the macrophage biomass reaction was set to 10% of the maximal flux of the viral biomass. Thus, the maximal fluxes for viral and macrophage objective functions changed from 0.027 & 0 1/h respectively for viral biomass optimization, into 0.0249 & 0.0027 1/h after

changing the weights and the upper limit. The same concept was implemented in the mouse model when adding the virus biomass objective function. We found a list of essential reactions for the macrophage and mouse models alone, reactions essential for both macrophage/mouse and virus and only in the case of macrophage-virus model 2 essential reactions only for the virus. These results are represented by the Venn diagram in (S1 Fig 2).

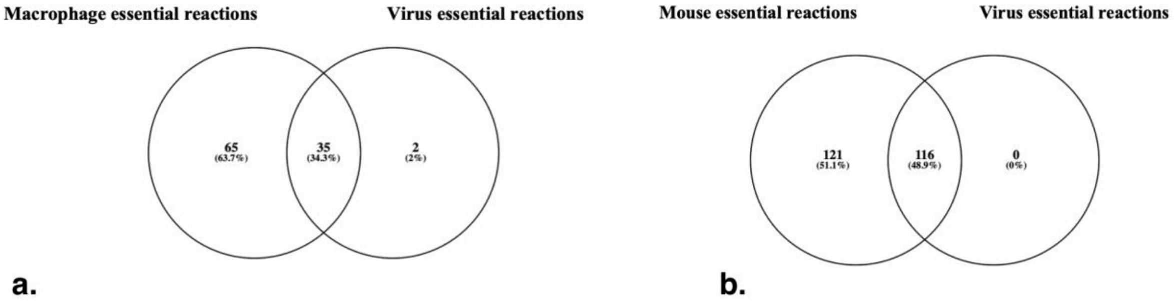

S1 Fig 2. Venn diagram representing the number of essential reactions for macrophage/mouse and for the virus. The intersection represents the number of essential reactions which are in common. **a.** 65 reactions are essential only for macrophage, 2 reactions are essential only for the virus and 35 essential reactions are shared. **b.** 121 reactions are essential only for mice, 0 reactions are essential only for the virus and 116 essential reactions are shared.

The 2 essential reactions for the virus in the macrophage-virus model are the phosphate exchange and guanosine monophosphate kinase (GK1). GK1 is involved in the purine metabolism that catalyses the reversible turnover of deoxyguanosine monophosphate (GMP) to deoxyguanosine diphosphate transferring a phosphoryl from ATP to GMP. In particular, GK1 is known as an essential building block of the RNA and for this reason an indispensable supplier for the construction of the RNA of the Zika virus. Using DrugBank (9), it was possible to identify some drugs that inhibit GK1, such as Acyclovir & Valacyclovir.

Conclusion

Constraint-based modelling is one of the most used approaches to model cellular metabolism. In this project, it was possible to obtain a host-virus integrated metabolic model using the human alveolar macrophage model *iAB-AMØ-1410* and the mouse model *iMM1415* as host cells and Zika as a virus. Single reaction deletion was used to identify potential targets such as GK1 for antiviral therapies. GK1 could play an essential role in different treatment strategies, but further analysis is needed to verify this finding.

References

1. Dick GWA, Kitchen SF, Haddow AJ. Zika virus. I. Isolations and serological specificity. Trans R Soc Trop Med Hyg. 1952 Sep;46(5):509–20.
2. de Araújo TVB, Ximenes RA de A, Miranda-Filho D de B, Souza WV, Montarroyos UR, de Melo APL, et al. Association between microcephaly, Zika virus infection, and other risk factors in Brazil: final report of a case-control study. Lancet Infect Dis. 2018 Mar;18(3):328–36.
3. Liu Y, Liu J, Du S, Shan C, Nie K, Zhang R, et al. Evolutionary enhancement of Zika virus infectivity in Aedes aegypti mosquitoes. Nature. 2017 May 25;545(7655):482–6.

4. Ikejezie J. Zika Virus Transmission — Region of the Americas, May 15, 2015–December 15, 2016. MMWR Morb Mortal Wkly Rep [Internet]. 2017 66. https://www.cdc.gov/mmwr/volumes/66/wr/mm6612a4.htm
5. Aller S, Scott A, Sarkar-Tyson M, Soyer OS. Integrated human-virus metabolic stoichiometric modelling predicts host-based antiviral targets against Chikungunya, Dengue and Zika viruses. J R Soc Interface. 2018 Sep 12;15(146).
6. Bordbar A, Lewis NE, Schellenberger J, Palsson BØ, Jamshidi N. Insight into human alveolar macrophage and M. tuberculosis interactions via metabolic reconstructions. Mol Syst Biol. 2010 Oct 19;6:422.
7. Duarte NC, Becker SA, Jamshidi N, Thiele I, Mo ML, Vo TD, et al. Global reconstruction of the human metabolic network based on genomic and bibliomic data. Proc Natl Acad Sci U S A. 2007 Feb 6;104(6):1777–82.
8. Sigurdsson MI, Jamshidi N, Steingrimsson E, Thiele I, Palsson BØ. A detailed genome-wide reconstruction of mouse metabolism based on human Recon 1. BMC Systems Biology. 2010 Oct 19;4(1):140.
9. Wishart DS, Feunang YD, Guo AC, Lo EJ, Marcu A, Grant JR, et al. DrugBank 5.0: a major update to the DrugBank database for 2018. Nucleic Acids Res. 2018 Jan 4;46(D1):D1074–82.

P5: Metabolic differences in high and low STAT3 expressing breast cancer - Sundas Arshad

Aim

This study aimed to compare the presence of metabolic pathways, reactions, metabolites, and genes in breast cancer with different STAT3 expression. Therefore, two context-specific models (high and low STAT3 expression) were built using as input a published model of a breast cancer cell line (MDA-MB-231) and patient expression data from the TCGA, previously segregated into high and low STAT3 expression.

Material and methods

To build two metabolic models of low and high STAT3 expression, we used a previously published breast cancer cell line model, *MDA-MB-231* (1) as a scaffold model. First, the model was constrained using DMEM medium (Dulbecco's modified Eagle's medium), which contains 20 components using the *constrain_model_rFASTCORMICS* function. In order to check for model consistency, i.e. remove all blocked reactions unable to carry a flux, FASTCC (included in the rFASTCORMICS pipeline) was run.

Patient gene expression data for breast cancer and healthy controls was taken from the TCGA (accession number: GSE62944). The RNA-seq data contains 1120 breast cancer samples and 22368 genes for each sample. Then, the breast cancer samples were stratified into high and low STAT3 expression status. Samples with a STAT3 gene expression below the 33rd and over the 66th percentile were selected for low and high expression of STAT3, respectively, leaving us with 749 samples for breast cancer.

In the next step, the gene expression data for low and high STAT3 expression was discretized into expressed, not expressed genes, and genes with unknown expression status with the *discretize_FPKM* function in rFASTCORMICS. Two consensus context-specific models were built by pooling the different samples of the high and low expression of STAT3 using rFASTCORMICS. Both models lost the biomass objective function during the model consistency check after the model building process.

Results

To get an overview on the models built for the high and low expression of STAT3, we compared their reactions, metabolites, and genes. There was no major difference in the number of reactions, metabolites, and genes observed between high STAT3 and low STAT3 models.

Model Fields	STAT3_high	STAT3_low
**Reactions**	889	934
**Metabolites**	748	779
**Genes**	990	949

S1 Table 4. Number of reactions, metabolites and metabolic genes in high and low STAT3 expression models.

The number of pathways observed for both models was similar (67 and 69 pathways in the high and low STAT3 expression model, respectively). However, there were two additional pathways (phenylalanine and vitamin B6 metabolism) in the low STAT3 expression model. No literature is available about a link between STAT3 and 'Phenylalanine metabolism', but there is a link between vitamin B6 and STAT3 expression that has been shown in previous studies (2). Vitamin B6 might have a general impact on the cellular management of stress by sensitizing a large panel of cancer cell lines to apoptosis (2). We also found the metabolite Glycero-3-phosphocholine which was exclusively present in patients with higher STAT3 expression. Recently, Glycero-3-phosphocholine was found to be negatively correlated with the flora bacteria *Faecalibacterium* in breast cancer patients. The abundance of this bacteria was reduced in breast cancer patients (3). Also, *Faecalibacterium* inhibits the production of IL-6 and phosphorylation of JAK/STAT3 cascade, thus preventing the metastasis and the proliferation of the breast cancer cells (3).

References

1. Yizhak, Keren et al. “A computational study of the Warburg effect identifies metabolic targets inhibiting cancer migration.” Molecular Systems Biology vol. 10,11 765. 1 Aug. 2014, doi:10.15252/msb.20145746
2. L. Galluzzi, I. V. (2012). Prognostic impact of vitamin B6 metabolism in lung cancer. *Cell reports 2012 Vol. 2 Issue 2* , Pages 257-269.
3. J. Ma, L. S. (2020). Alter between gut bacteria and blood metabolites and the anti-tumor effects of Faecalibacterium prausnitzii in breast cancer. *BMC microbiology Vol. 20*, Pages 1-19.
